# Review of Semantic Segmentation of Medical Images Using Modified Architectures of UNET

**DOI:** 10.3390/diagnostics12123064

**Published:** 2022-12-06

**Authors:** M. Krithika alias AnbuDevi, K. Suganthi

**Affiliations:** Vellore Institute of Technology, Chennai 600127, India

**Keywords:** UNET, semantic segmentation, dice similarity coefficient, CNN, MRI

## Abstract

In biomedical image analysis, information about the location and appearance of tumors and lesions is indispensable to aid doctors in treating and identifying the severity of diseases. Therefore, it is essential to segment the tumors and lesions. MRI, CT, PET, ultrasound, and X-ray are the different imaging systems to obtain this information. The well-known semantic segmentation technique is used in medical image analysis to identify and label regions of images. The semantic segmentation aims to divide the images into regions with comparable characteristics, including intensity, homogeneity, and texture. UNET is the deep learning network that segments the critical features. However, UNETs basic architecture cannot accurately segment complex MRI images. This review introduces the modified and improved models of UNET suitable for increasing segmentation accuracy.

## 1. Introduction

**Figure 1:**
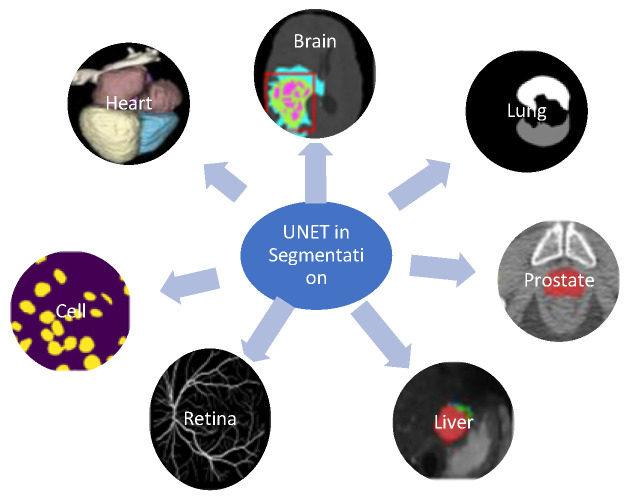
Application of UNET in medical image segmentation.

Principal component analysis [1], fuzzy c-means Hsieh [2], Gabor filter [3], and multilevel fuzzy c-means [4] are examples of traditional machine learning techniques. However, the performance of these algorithms in the field of computer vision is not sufficient. Therefore, deep learning is now widely employed in various industries [5,6,7,8,9,10,11,12,13], for example, to tackle problems in computer vision and succeed in image recognition. Deep learning techniques are used to assess complex and diverse pathological images. Deep learning techniques can learn coarse and fine representations in all layers and perform end-to-end learning. There are the following two basic frameworks: CNN and the FCN for segmentation. Convolutional neural networks (CNN) perform well in classifying images and significantly improve segmentation. Initially, the categorization of image patches was a widely used deep learning approach where each pixel was sorted into matching categories separately by employing image blocks around each pixel. On the other hand, the FCN framework expands the fundamental CNN structure without a fully connected layer to enable intensive prediction in medical image processing. The problem of pixel location is solved using the shallower high-resolution layer, while the issue of pixel categorization is solved using the deeper layer. This structure is used in almost all current medical image semantic segmentation research. The internal structure of the human body is extremely complex. Hence, it is difficult for doctors to determine the disease’s severity and location. Many approaches have been developed to overcome this challenge, and new research is constantly developing more novel and innovative methods. With the widespread adoption of image-aided medical diagnosis, segmentation is the desired process in medical image analysis. This is supported by the large number of papers explicitly published for the segmentation process, in which U-net survive prominent method [14,15]. UNET can improve the efficiency of segmenting disease-affected regions of the brain, lung, retina, liver, etc., as depicted in Figure 1.

**Figure 2:**
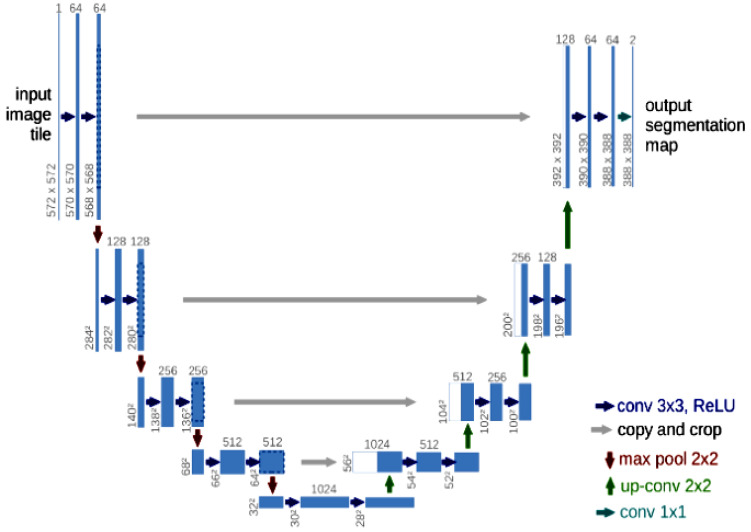
UNET model [16].

Semantic segmentation is the classification of features in images based on pixels. Due to the lack of image detail, it is impossible to derive precise boundaries using image semantic feature information. The UNET model [16] designed by Olaf Ronneberger, Philipp Fischer, and Thomas Brox is shown in Figure 2, an ideal solution for medical image segmentation tasks, it efficiently uses the skip connection to merge feature maps of low-resolution and high-resolution images [17]. UNET is the CNN framework; it has a simple encoder and decoder network shaped like a U. This model can be well-trained with fewer samples. Despite the small training dataset, it provides precise segmentation results. The features were learned optimally using a UNET-based model.

The survey articles [18,19] are related review works in which the application of UNET in various imaging modalities and UNET variants used in medical image segmentation are discussed. Our survey provides an

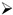

In-depth review of UNET-modified architectures;

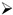

Benchmark datasets and semantic architectures specifically designed for medical image segmentation;

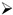

Presents the application of modified architectures of UNET in the segmentation of anatomical structures and a lesion in different organs to diagnose diseases;

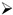

An updated survey of the improvement mechanisms, latest techniques, evaluation metrics, and challenges.

## 2. Study Method

**Figure 3:**
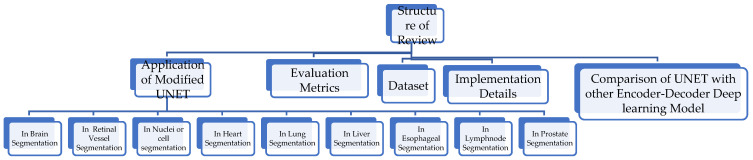
Structure of the review.

The references are taken between the time frame of from 2015 to 2022. This survey is confined to the application of modified architectures of UNET in biomedical image segmentation. To determine the relevant quality of the paper, the references are taken from peer-reviewed journals. All architectures are thoughtfully collected from the original paper with a unique model focusing on enhancing accuracy and reducing complexity. Managing and comprehending the database format is a difficult task for researchers. Hence, this survey includes a separate section describing the medical image analysis database. It explains the benefits of adding the networks to the UNET in segmenting the lesion and tumor from different organs using images from imaging modalities. The structure of this review is given in Figure 3.

## 3. Application of Modified UNET

This section highlights the modified architecture of UNET for segmenting the region of interest from different imaging modalities to identify the severity of diseases.

### 3.1. InBrain Segmentation

#### 3.1.1. UNET with Generalized Pooling

This model modifies the pooling operation to enhance segmentation [20]. In the CNN and FCN models, the dimension is reduced to address the overfitting issue via max pooling or average pooling.Features are not precisely defined for variable data in down-sampling. A brain tumor’s characteristics are very minute, so it is vital to minimize feature loss. A new generalized pooling (GP) method was developed to extract more prominent features from downsampling and improve segmentation performance. This approach adapts a pooling kernel’s weights based on the input MRI images or feature maps. The initial average weight α_0_ of each element is assigned as in Equation (1). The mean is given in Equation (2) as follows:

(1) $$\alpha 0_{\;}=\frac{1}{p\times q}$$

where *p* is the length and *q* is the width of the pooling kernel.

(2) $$\hat{z}\frac{\sum_{r=1}^{p}\sum_{s=1}^{q}z_{rs}}{p\times q}$$

#### 3.1.2. Stack Multi-Connection Simple Reducing Net (SMCSRNET)

**Figure 4:**
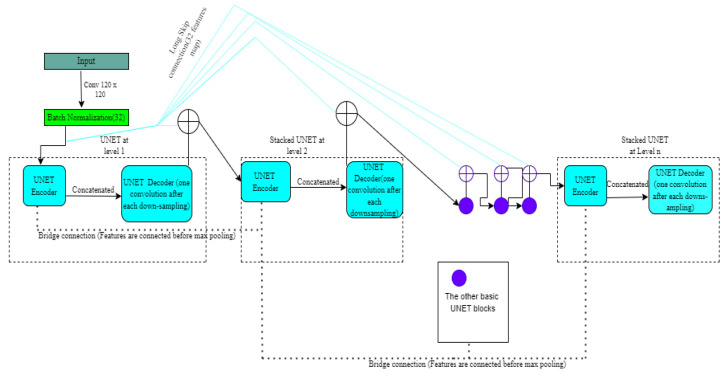
The architecture of SMCSRNET.

Multi-connection stack, a novel framework known as simple reducing net (SMCSRNet) [21], is constructed using certain fundamental building elements (SRNet). Four down-sampling/up-sampling procedures were carried out throughout the encoding/decoding phases.UNET was further improved to better suit stacking to segment brain tumors. There is only one convolution process before each down-sampling. The processes of cropping and copying are maintained between decoding and encoding. This design aims to reduce parameters and simplify the network structure. It is important to note that the SMCSRNet model requires significantly less training time than the stacked UNET. In addition, the precision of this model has increased. The final block contains 32 feature maps stacked to the input image using the long skip connection depicted in Figure 4.

#### 3.1.3. 3D Spatial Weighted UNET

**Figure 5:**
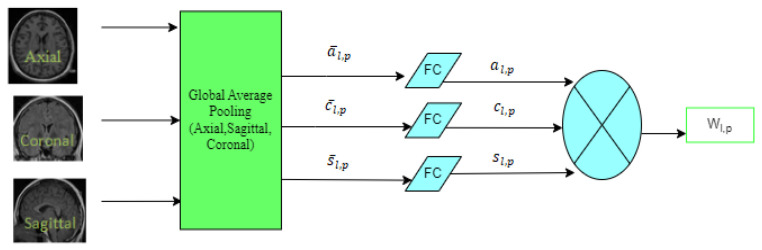
WorkFlow of VFR.

To properly utilize spatial contextual data at the intra-level plane and apply it to volumetric spatial weighting at the inter-level plane, the volumetric feature recalibration layer (VFR) is added to a 3D spatially weighted UNET [22]. It extracts geographic statistical information. The spatial information is compressed using global average pooling. The VFR is incorporated in this model before the de-convolutional layer and the max pooling layer in the encoder and decoder, respectively. Prior to resizing, it can be used to improve the features to prevent the loss of spatial information. Spatial statistical information is obtained by applying the global average pooling operation in each plane in Equation (3). The entire plane’s spatial information is multiplied by the tensor product term to form the lower-weight tensor and change the weights of the volumetric input information. The workflow of VFR is shown in Figure 5.

(3) $$\begin{array}{c} {\bar{a}}_{lp}=GAP_{a}\left(f_{l},p\right)=\frac{1}{IJ}\sum_{ij}f_{l,p}\left(i,j,k\right),\\ {\bar{c}}_{lp}=GAP_{c}\left(f_{l},p\right)=\frac{1}{IK}\sum_{ik}f_{l,p}\left(i,j,k\right),\\ {\bar{\;s}}_{l,p}=GAP_{s}\left(f_{l},p\right)=\frac{1}{JK}\sum_{jk}f_{l,p}\left(i,j,k\right). \end{array}$$

where *f_l_* is the volumetric feature tensor input to the first VFR layer, *i* is the length, *j* is the width, *k* is the height, and *p* channels. The statistical information in three planes (axial, coronal, and sagittal) are ${\bar{a}}_{lp},\;{\bar{c}}_{lp}\;\mathrm{and}\;{\bar{s}}_{lp}$. The weighted feature tensor is mathematically given in Equation (4) as follows:

(4) $$w_{l,p}=a_{l,p}⊗c_{l,p}⊗s_{l,p}$$

This model is extended to the multimodality images with feature tensor values three times higher than for a single modality.

#### 3.1.4. Anatomical Guided UNET

**Figure 6:**
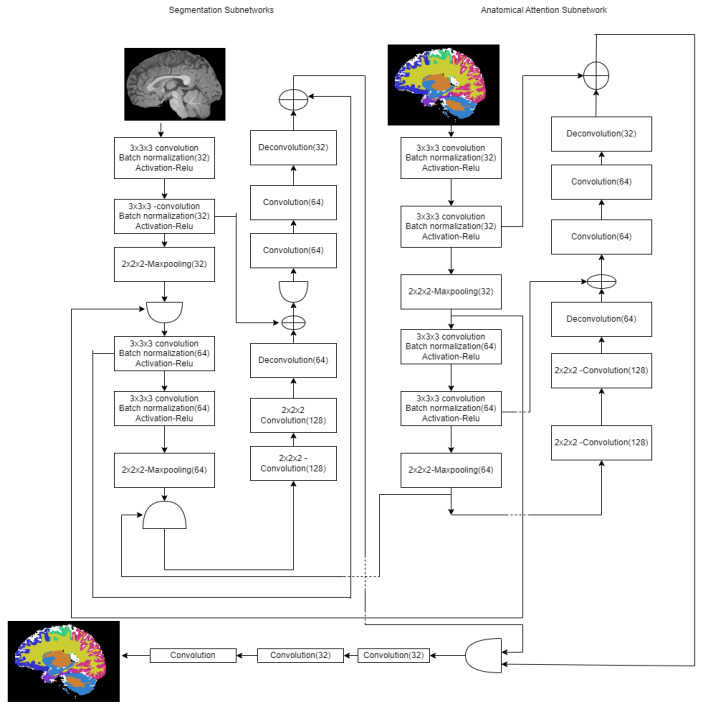
Anatomical attention guided model.

The segmentation and the anatomical attention sub-networks are the two sub-networks used in this model [23]. The segmentation network provides the local contextual information and learns the feature map from the image intensity. The anatomical images in the atlases train the anatomical networks. This anatomical gated network guides the segmentation network to segment the appropriate region of interest. The proposed anatomical guided architecture UNET is laid out in Figure 6. This work uses an anatomical gate to combine the features created by two sub-networks.

The feature maps [$f_{i}^{s}\;\left(\mathrm{feature}\;\mathrm{map}\;\mathrm{from}\;\mathrm{segmentation}\;\mathrm{network}\;\mathrm{in}\;\mathrm{the}\;\mathrm{sth}\;\mathrm{network}\right),f_{a}^{s}$ (feature map from anatomical attention subnetwork)] are concatenated channel-wise. It is fed into two convolutional layers (size: 1 × 1× 1), and a non-linear sigmoid unit follows each convolutional layer to learn the weight tensor. (e.g., $o_{i}^{s}$) for each input feature map. The learning mechanism of weighted tensor is given in Equation (5) as follows:

(5) $$\begin{array}{c} o_{i}^{s}=\sigma \left(W_{i}^{s}[f_{i}^{s},f_{a}^{s}\}+b\right),\\ o_{a}^{s}=\sigma \left(W_{a}^{s}[f_{i}^{s},f_{a}^{s}\}+b\right).\;\; \end{array}$$

The anatomical gate, feature map output ($f_{o}^{s}$) is given by the following:

(6) $$f_{o}^{s}=o_{i}^{s}\;\cdot f_{i}^{s}+o_{a}^{s}\;\cdot \;f_{a}^{s}$$

The anatomical attention gate contains brain structure information provided by multiple atlases at different scales. This model automatically learns the optimal weights generated by the two subnetworks and efficiently fuses the two subnetworks for accurate ROI segmentation.

#### 3.1.5. MH-UNET

**Figure 7:**
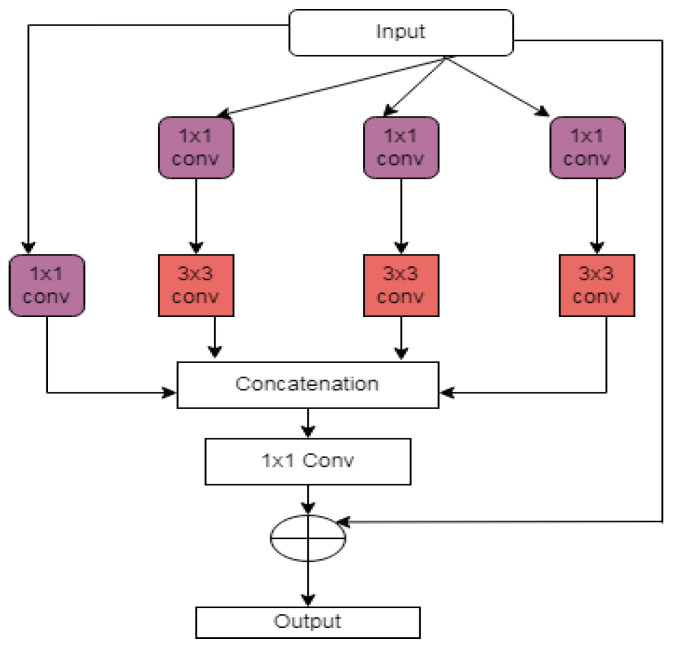
Residual inception block.

**Figure 8:**
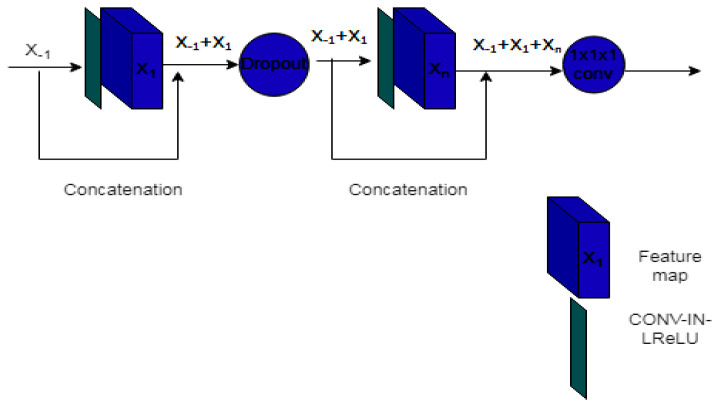
Dense block.

In multi-scale UNET [24], several dense blocks, residual inception blocks, and hierarchical blocks are included in the decoder and encoder, which reduce the trainable parameters. Residual inception blocks (in Figure 7) extract valuable features. It learns much global and local information from a large receptive field.Residual inception block output is given in Equation (7).

(7) $$y_{l+1}=\left(\left(f_{one}\left(f_{d}\left(y_{l}\right)⊚y_{l}\right)\right)⊕f_{one}\left(y_{l}\right)\right)$$

where $y_{l}$ is the output of current layer, *f_d_*(.) is for Dilated Conv-IN-LeakyReLU, *f_one_* is for 1 × 1 × 1 Conv-IN-LeakyReL. The hierarchical block extracts multi-scale information features. In the hierarchical block, dilated convolutional layers increase the receptive field without increasing the dimensions. On the other hand, a dense network (in Figure 8) decreases the trainable parameter and redundant feature for 3D convolution. The working condition of a dense block is described in Equation (8).

(8) $$x_{l+1\;}=g\left(x_{l}\right)ʘ\;x_{l}$$

where *x* is the output of the current layer and *g* represents the flow of Conv-IN-LeakyReLU and ʘ is the concatenation function. Deep supervision is also proposed for superior segmentation accuracy and faster convergence.

#### 3.1.6. MI-UNET

**Figure 9:**
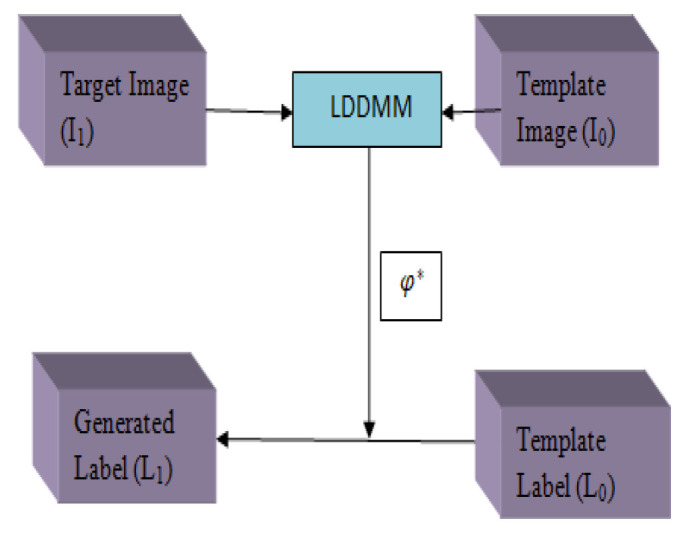
MI-UNET architecture.

In MI-UNET [25], brain parcellation information is obtained for the input MRI, and this information is additionally given as the input to the UNET (shown in Figure 9). LDMM [26] image registration algorithm is used for extracting the segmentation details from the atlas-based registration, and the MRI image is segmented into GM, WM, and LV.

The brain parcellation is obtained as follows:

(9) $$L_{1}=L_{0}⊚\Phi_{a}^{*}$$

In Equation (9), *L*_1_ is the brain parcellation, *L*_0_ is the template label and $\Phi_{a}^{*}$ is the transformation. The GM, WM, and LV parcellation are obtained using atlas-based segmentation, which is independent of the subsequent deep learning-based stroke lesion segmentation.

#### 3.1.7. Multi-Res Attention UNET

In multi-res attention gate UNET [27], Multi resnet [28] block reduces the filter dimension by splitting the 5 × 5 and 7 × 7 into the series of 3 × 3. In addition, two-layer filters (*L*1, *L*2) are implemented to reduce the requirement of high memory. *L*1 and *L*2 filter parameters are given in Equations (10) and (11), respectively.

No of the filters parameter in *L*1 *= k*^2^
*× n × l* (10)

No of the filter parameter in *L*2 *= (k^’^)*^2^
*× l*^2^ (11)

A residual path is added to overcome the semantic gap problem between the encoder and decoder.

(12) $$\mathrm{Res}x_{\;}=\theta_{X_{3\times 3}}.{µ}_{i}+w_{X_{1\times 1}}({µ}_{i})+b_{x}$$

(13) $$\mathrm{Res}y_{\;}=\theta_{Y_{3\times 3}}.{µ}_{i}+w_{Y_{1\times 1}}({µ}_{i})+b_{y}$$

In Equations (12) and (13), variable x represents the first layer, and variable y represents the second layer. Whereas θ is the filter term, µ_i_ is the feature map, w is the convolution, and b is for bias. The attention-gating block has the GS(gating signal). This signal guides the attention block to choose the exact features. Extracted spatial information is passed through a 1 × 1 ($w_{GS}$) convolution operation. Finally, a ReLU activation function is applied to the output. As shown in Equation (14), the resulting signal is the attention-gating signal.

(14) $$\mathrm{GS}=\;ReLU\left(w_{GS}\left(s\right)+b_{GS}\right)$$

### 3.2. In Retinal Vessel Segmentation

#### 3.2.1. GLUE [29]

A weighted U-Net (WUN) and a weighted residual U-Net(WRUN) form this model. The WUN first creates a coarse segmentation map using patches that have been globally improved. The WRUN then enhances the locally upgraded patches, whose parameters are automatically updated rather than adjusted. Discriminative features are obtained by adding residual connections to the second half of the model (WRUN). Additionally, it uses the cascaded U-Net structure, which stands to gain improvements in retinal imaging both locally and globally. On retinal images, the contrast-limited adaptive histogram equalization (CLAHE) operation [30] is used to increase contrast.A circular template mask for the region of interest is created to obtain the location of the fundus. This mask can be used as the weighted attention mask to segment only the fundus and leave the irrelevant area. The weighted attention mask is multiplied by the feature map of the last WRUN layer, and the skip connection improves the depth and accuracy of UNET. It is implemented as in Equation (15).

(15) $$y=F\left(x,\left\{w_{i}\right\}\right)+H\left(x\right)$$

where *x* represents the input, *H* represents the identity mapping function and $\;w_{i}\;\mathrm{represents}\;\mathrm{the}$ weight.

#### 3.2.2. S-UNET

**Figure 10:**
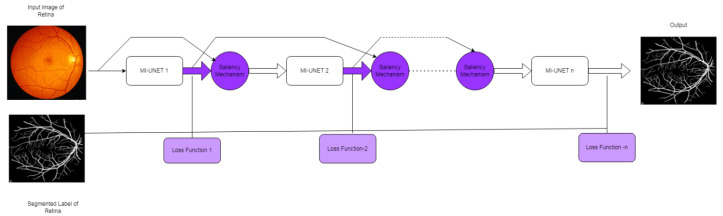
S-UNET.

The minimum UNET is the foundation of the salient UNET [31] architecture. The network parameter can be decreased from 31.03 M to 0.07 M with minimal UNET. The bridge-style architecture, with two Mi-UNETs cascading, provides a prominent mechanism. Some features were taken from the first MI-UNET and provided as foreground attention directions for the next MI-UNET (shown in Figure 10). Features from all the output units are concatenated with the input block. It is given in Equation (16).

O_1_ = W_1 × 1_ (16)

**Figure 11:**
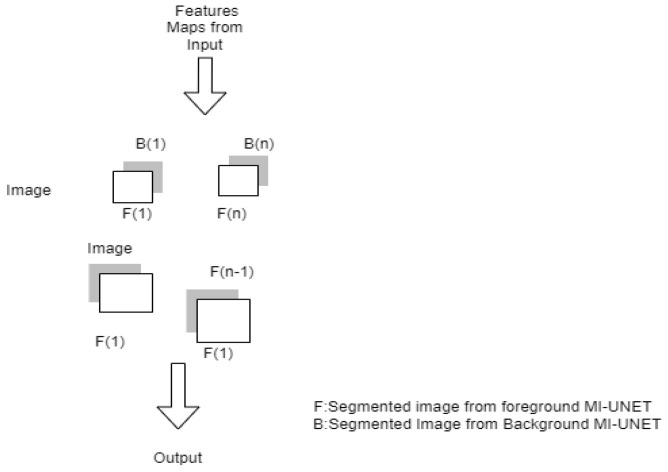
Saliency mechanism.

The saliency mechanism is shown in Figure 11 and defined in Equation (17).

(17) $$\mathrm{sO}1_{\;}=(W_{1}X{)}_{f}⊕X_{1}$$

From Equation (17), it is clear that the second minimal UNET gets the enhanced input.

### 3.3. In Nuclei or Cell Segmentation

#### 3.3.1. As-Unet

**Figure 12:**
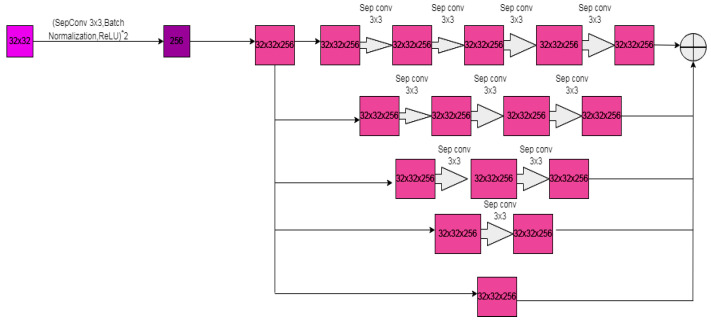
Part of AS-UNET.

The atrous convolution is added between the encoder–decoder to increase the network’s receptive field without affecting the image resolution. Atrous convolution can change the convolution step for multi-scale information. The 3 × 3 Separable convolutional is added with the ReLu activation function. There are 4 dilation rates, and 5 parallel and cascade atrous separable convolutions are added, and it is shown in Figure 12. The size of the AS-UNET [32] model, the number of trainable parameters, and the evolution time decreases using separable convolution. In AS-UNET, log-Dice loss and the focal loss are added to calculate the loss function as in Equation (18).

(18) Loss=λ*logDL+(1−λ)*FL

In Equation (18), LogDL = −log(2*($y_{t}\cap y_{p}))/\left(\left|y_{t}\right|+\left|y_{p}\right|\right)$ is the logDice loss and FL = $y_{t}*\log\left(y_{p}\right)*{\left(1-y_{p}\right)}^{\gamma }$ is the focal loss, *y_t_* is the GT value, *y_p_* is the predicted value, and λ is the training parameter.

#### 3.3.2. RIC-UNET

**Figure 13:**
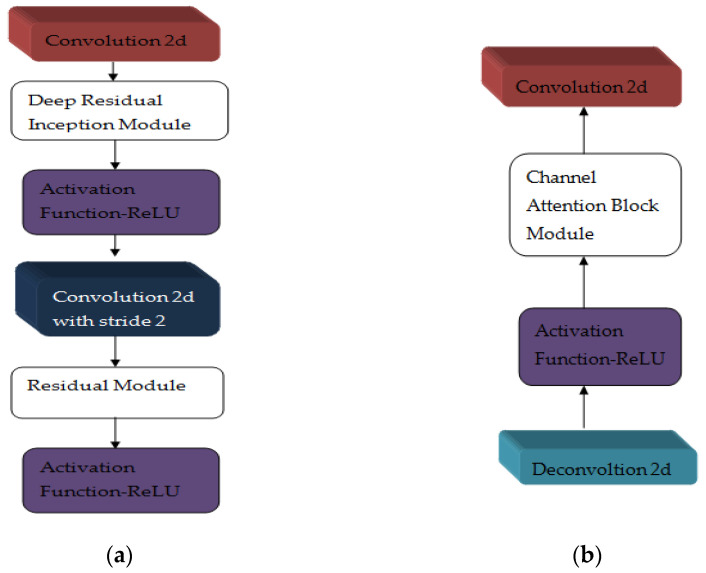
(**a**) RI Block (**b**) DC Block.

The multi-scale residual inception block and channel gate are applied in RIC UNET [33]. The residual inception block extracts the multi-scale feature information. Cell contour obtained from this network is used to segment the dense cell and reduce the cell level error. The channel attention block selects the high-resolution features with the low-resolution information taken from the up-sampling process. The structure of the RI block and DC block is laid out in Figure 13.

### 3.4. UNET in Heart CT Segmentation

#### 3.4.1. Modified 2D UNET

**Figure 14:**
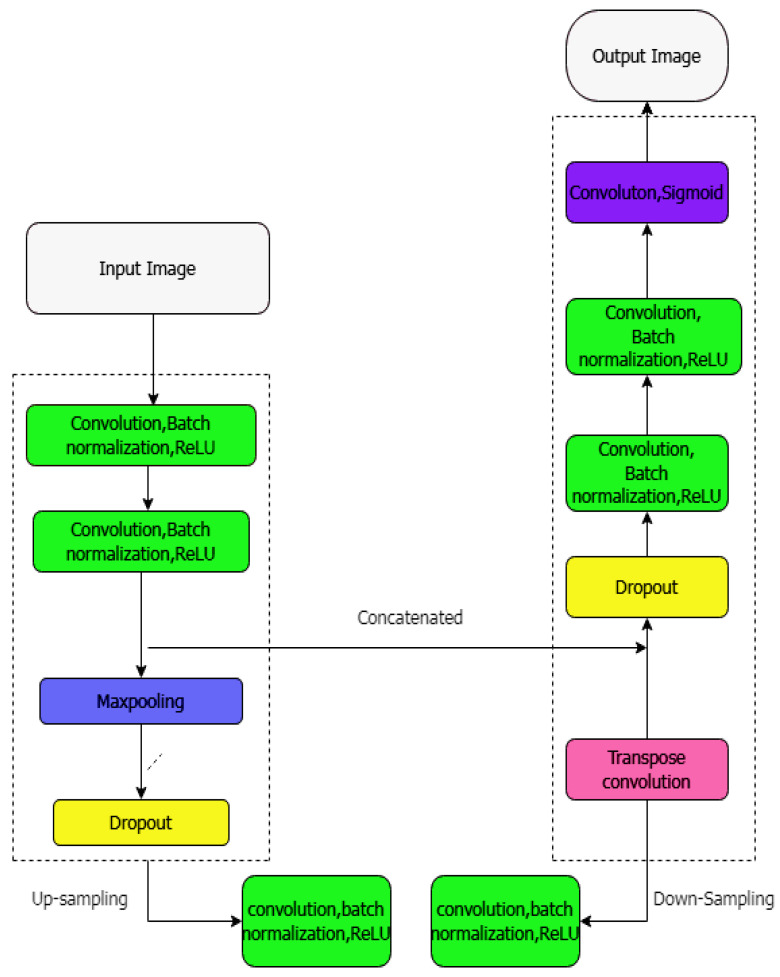
The architecture of adding batch-normalization and dropout layer in up-sampling and down-sampling processes.

A modified 2D UNET model [34] is the next-level model of the fundamental 2D UNET model. It adds a dropout and batch normalization before each convolution block (depicted in Figure 14) to segment the aorta and coronary artery. The internal covariate shift affects the training process. The batch normalization stabilizes the training by normalizing the inputs for each mini-batch, which was achieved by ciphering the standard deviation and mean of each input variable for the layer of a single mini-batch.By randomly setting the weights to zero, the over-fitting was reduced using the dropout layer.

#### 3.4.2. UCNET with Attention Mechanism

A negative mining technique is used in this model [35] to suppress the uninterested area. First, the number of negative sample examples *N_s_* for each training sample was estimated using Equation (19).

(19) $$N_{s}=N_{n}\max\left(2N_{p},\frac{N_{n}}{8}\right)$$

In Equation (19), *N_s_* is the number of negative samples, and *N_p_* is the number of positive samples.

**Figure 15:**
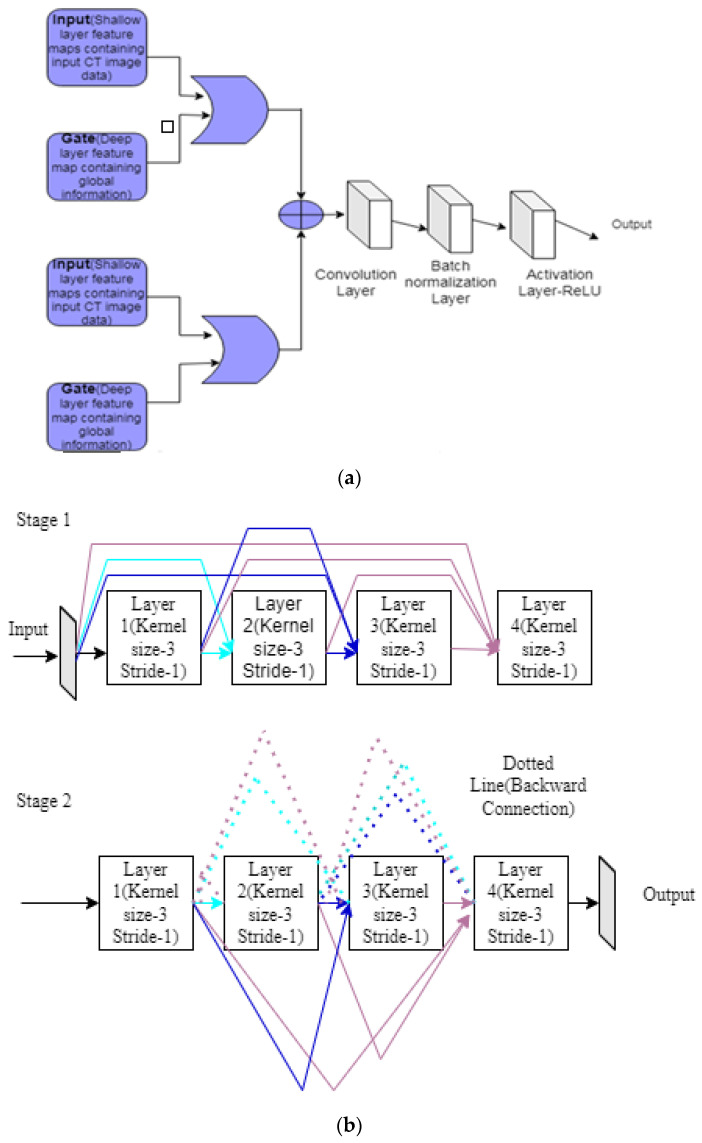
(**a**) Attention mechanism (**b**) U-clique NET.

The attention mechanism and U-clique net focus only on the vital region. In the attention mechanism, input is in the shallow layer, and the gate uses the deep layer. Both are added to generate the attention map (Figure 15a) and are given to convolutional block, batch normalization, and RELU. U-clique UNET is laid out in Figure 15b. In stage 1, each layer is connected with the previous layer to update the next layer. In the next stage, layer 2 is concatenated to layer 1 in a forward direction, and the third and fourthlayers in the feedback directly to stage 1. This process will improve communication between the layers. Finally, heart regions are divided into segments, and the Jaccard score is calculated.

### 3.5. UNET in Lung Segmentation

#### 3.5.1. Cascaded UNET [36]

The network includes the EM (expectation maximization) framework [37] to account for the prior function of the disease-affected area. UNET is initially fine-tuned to discover the consolidated region from the labels at the patient level by applying the EM algorithm after being trained with labeled, segmented image of the region of interest. Then, the latent variable y is solved pixel-wise with the EM algorithm given in Equation (20).

(20) $$y_{ij}=\{\begin{array}{c} 1,|\;f_{j}\left(x_{i};\theta^{\prime }+\varphi \left(z_{i},x_{ij}\right)\right)>1\\ 0,\;\;Otherwise \end{array}$$

#### 3.5.2. Res-D-UNET

**Figure 16:**
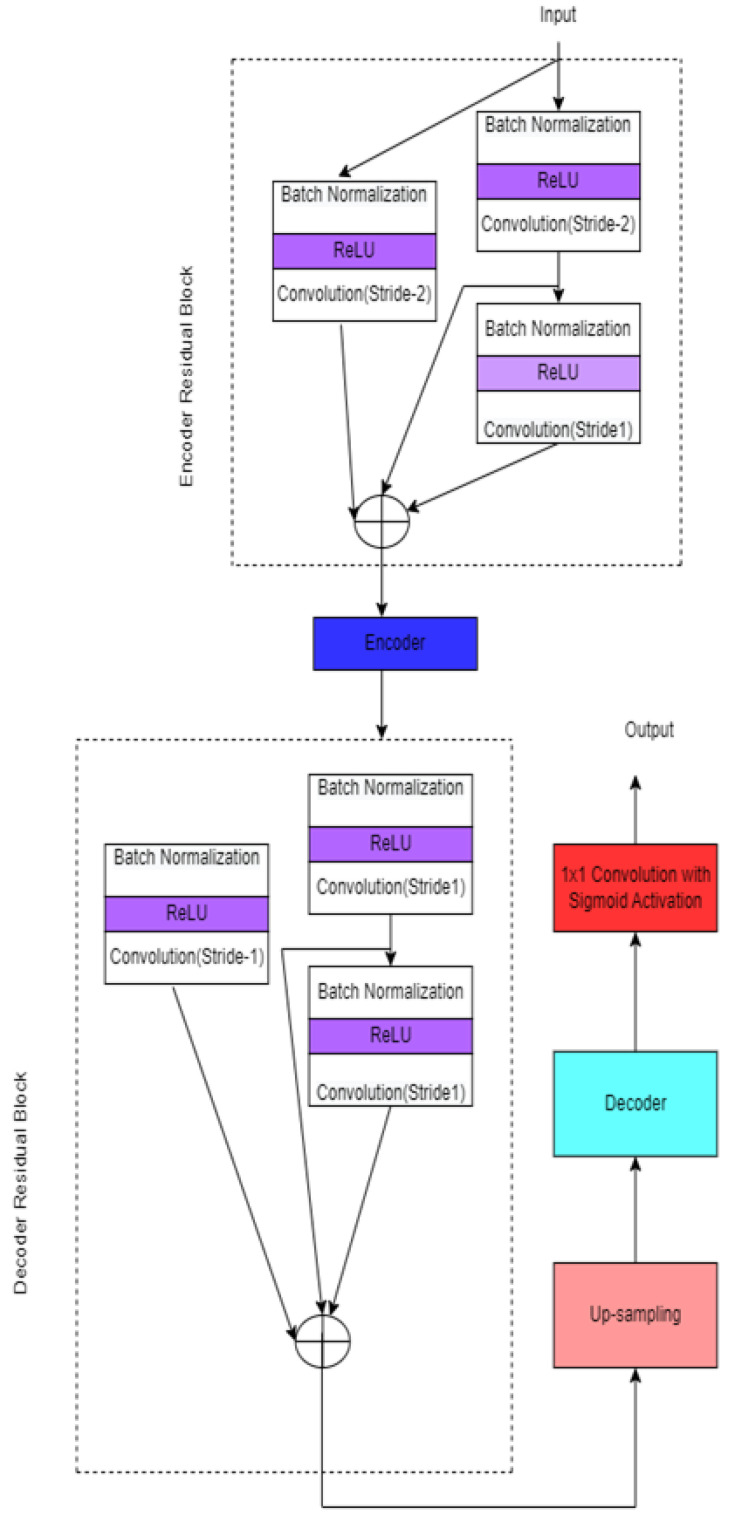
Res-D-UNET.

Res-D-UNET [38] extracts all the high-level features from the intra-slice plane. An overview of a residual dense block is shown in Figure 16. The exclusive feature from the top layer to the bottom layer gets utilized; hence, vanishing gradient problem is reduced during the training period of the network.Binary cross entropy, similarity index, and dice loss are the loss functions calculated in this model.

A ReLU activation layer, a batch normalization layer, and two convolution layers with strides of 2 and 1 are included in each convolution block. In addition, a convolutional layer connects encoder input and output with a stride of 2, and a BN layer is used in identity mapping.

### 3.6. UNET in Liver Segmentation

**Figure 17:**
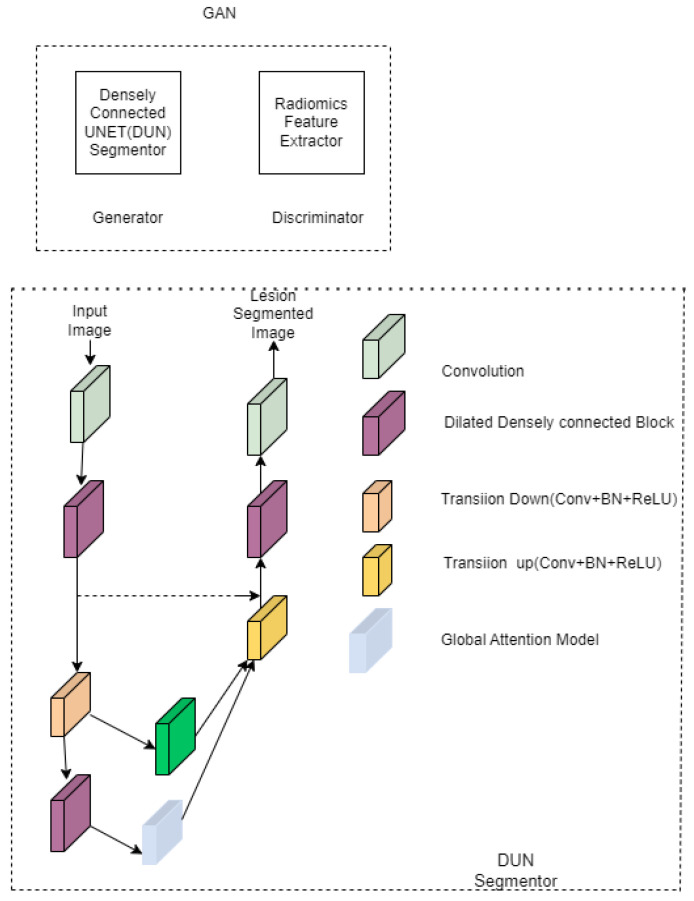
Densely connected UNET.

Multi-phase dynamic contrast enhancement MRI radiomics features [39] insist on extracting the ICR characteristics from non-contrast images. Therefore, it is carried out without the use of contrast chemicals. In this work [40], the radiomics features guide UNET and generational adversarial network. Radiomics features are used at the discriminator, and the DUN (shown in Figure 17) is used as the segmenter at the generator network. UNET disseminates the directed knowledge. The gradient disappearance is reduced by combining a dilated and densely convolutional network. A global attention model extracts the desired characteristics from the pixels in low-contrast images. The discriminator of the GAN receives the MCRF (multi-phase radiomics feature) as input, which easily separates lesions from non-contrast images. Radiomics and semantic feature extraction models are connected with radiomic-guided layer connections at the discriminator. Semantic features are extracted using VGG 16 [41]. PyRadiomics [42] is an open-source tool to extract the features from the MRI.

### 3.7. UNET in Esophageal Segmentation

**Figure 18:**
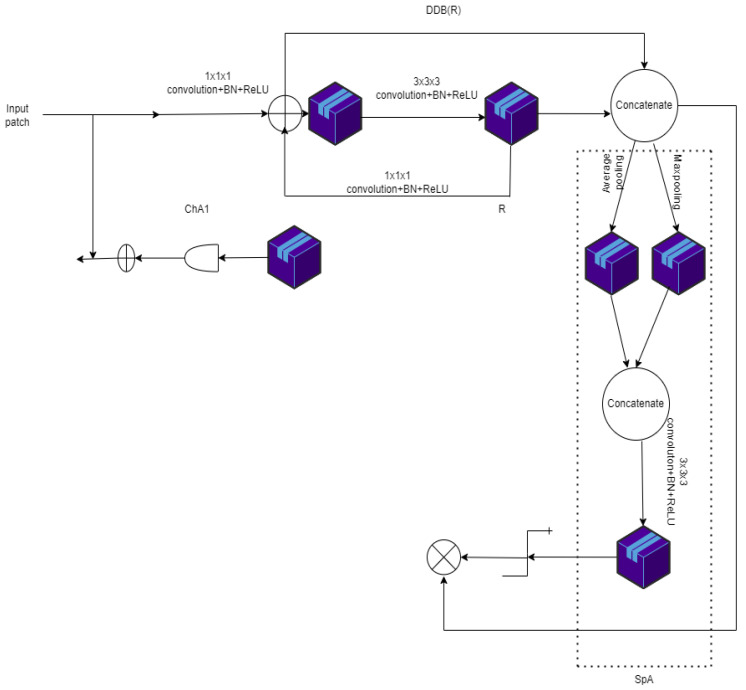
Architecture of DDAUNET.

In this model of a dilated dense block, channel attention (CHA1) and spatial attention (SPA) gates are used. The spatial gate retrieved tumor features in the main block were retrieved by the spatial gate. In the space between the paths of extracting and contracting, the channel gate filtered out the unimportant features.Dubbed dilated dense attention UNET model [43] (DDAUNET), it segments the esophageal GTV (gross tumor volume). Its architecture is shown in Figure 18.

Figure 18 denotes DDSCAB (dilated dense spatial and channel attention block) and DDB (dilated dense block). R represents the number of sub-DDBs. For example, chA1 is a skip connection channel attention gate, ChA2 is a DDSCAB block channel attention gate, and SpA is a DDSCAB block spatial attention gate. Although ChA1 is not included in the final network (DDAUnet), it is used in some of the experiments.

### 3.8. UNET in Lymphnodes Segmentation

**Figure 19:**
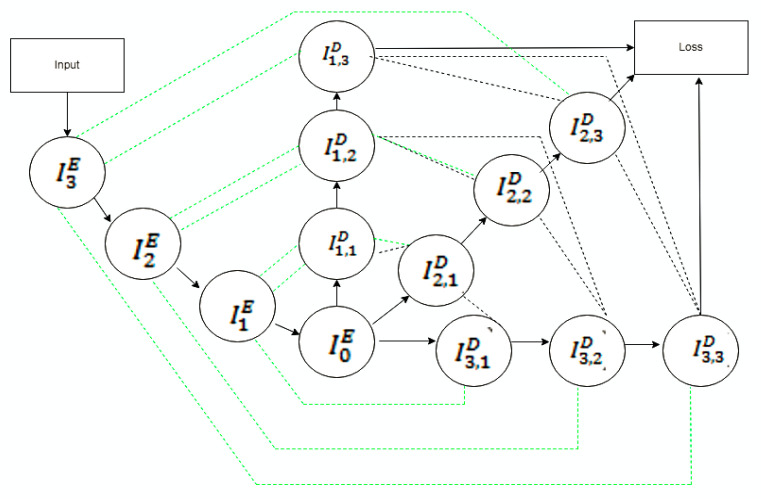
RDS UNET.

The body has lymph nodes and lymphoid tissues in all parts, making it challenging to distinguish lymphoma on a full-body CT scan. Hyperdense encoding using UNET architecture and recurrent dense Siamese decoding is employed in this model [44] at the encoder and decoder, respectively. The segmentation accuracy is increased using bootstrapping in re-sampling and a stable-gradient adaptive similarity dice loss function. The recurrent dense Siamese UNET in Figure 19 enables the spatial and temporal correlation. The Siamese decoder has two similar subnetworks for generating the feature vector for the input and eradicating the duplicate features.

### 3.9. UNET in Prostate Segmentation

**Figure 20:**
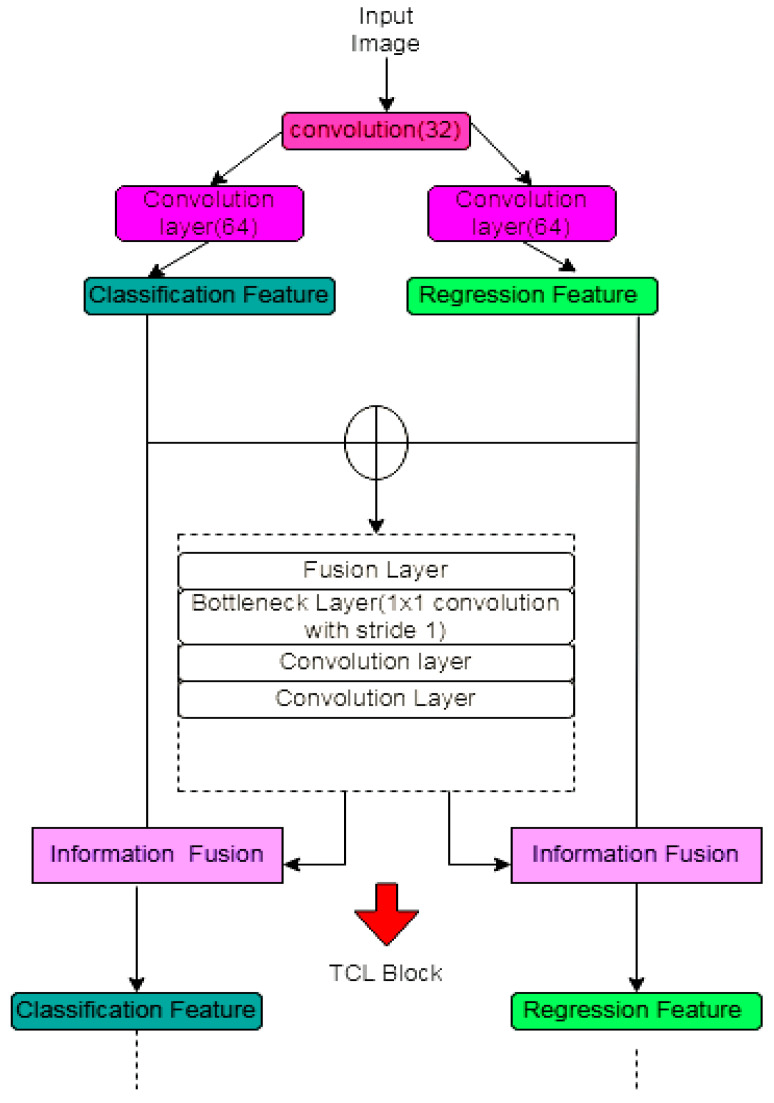
Task consistency block (TCL).

A challenging task in prostate segmentation is (1) fast localization of the prostate boundary and (2) accurate segmentation. Hierarchically fused UNET is the multitask FCN. Adding an attention-based task consistency learning (TCL) module allows the encoder and decoder to share task-related knowledge. This research [45] implements a channel-based and a position-based attention network to learn the best information (shown in Figure 20). 

## 4. Evaluation Metrics

- DSC

The dice similarity coefficient (DSC) was first proposed by Dice [46]. It uses a reproducibility validation metric and an index of spatial overlap. Fleiss also referred to it as the percentage of explicit agreement [47]. DSCs values range from 0 to 1, which denotes the entire spatial similarity between two data sets from binary segmentation, indicating total spatial overlap. It predicts the similarity index between the ground truth and the predicted image by comparing the pixel-wise agreement between the two images.

(21) $$\mathrm{DSC}=\frac{2*|X\cap Y|}{\left|X|+|Y\right|}$$

In Equation (21), DSC is the dice similarity coefficient, *X* is the ground truth image pixels, and *Y* is the predicted image pixels. It should be higher.

- PPV–positive predictive value or precision

It measures the precision of prediction [48,49,50,51,52] by counting the number of actual samples. It is formulated in Equation (22).

(22) $$\mathrm{PPV}=\frac{TP}{TP+FP}$$

- Accuracy

Accuracy calculates the correctly classified pixels in the images. The formula for the accuracy is given in Equation (23).

(23) $$\mathrm{Accuracy}=\frac{TP+TN}{TP+FN+TN+FP}$$

- Sensitivity or recall

It measures [53,54] the number of false and true images. It is otherwise known as the positive rate. The calculation of recall is given in Equation (24).

(24) $$\mathrm{Sensitivity}=\frac{TP}{TP+FN}$$

- F1 score

This metric [55] gives the balance value in-between precision and recall. The result of 1 represents the best prediction. F1 score is formulated in Equation (25).

(25) $$F1=2\times \frac{precision\times recall}{precision+recall}$$

- AUC (area under curve) [56]

It is the plot of the receiver under the operation curve according to the true positive rate(TPR) at the vertical axis and false positive rate(FPR) at the horizontal axis. TPR and FPR are given in Equations (26) and (27), respectively.

(26) $$\mathrm{TPR}=\frac{TN}{TN+FP}$$

(27) $$\mathrm{FPR}=\frac{FP}{FP+TN}$$

- The 95th percentile Hausdroff distance

Hausdroff distance [57] is the prediction of the distance between prediction and ground truth images. Small value of HD represents the high segmentation accuracy.

(28) $$\mathrm{HD}(S,L)=\max\{k^{th}{}_{s\in S}min_{g\in G\|S-L\|},\{k^{th}{}_{g\in G}min_{s\in S\|L-S\|}\}$$

In Equation (28), *S* is the segmented image, and G is the ground truth image.

- Absolute volume difference

It predicts the difference between segmentation and label in terms of volume. A smaller range of AVD [58] gives better segmentation.

(29) $$\mathrm{AVD}(S,L)=\frac{V_{s}-V_{L}}{V_{L}}\times 100\%$$

In formula (29), *V_s_* is the volume of the segmented image, and *V_L_* is the volume of the labeled image.

- Jaccard score or IOU [59]

(30) $$\mathrm{Jaccard}(A,B)=\frac{\left|A\cap B\right|}{\left|A\right|+\left|B\right|-\left|A\cap B\right|}$$

In Equation (30), *A* is the ground truth, and *B* is the segmented image.

- Matthews correlation coefficients (MCC) [60]

It is a statistical tool to identify the difference between predicted and actual images, which Brain Matthew formulated.

(31) $$\mathrm{MCC}=\frac{TN\times TP-FN\times FP}{\sqrt{\left(TP+FP\right)\left(TP+FN\right)\left(TN+FP\right)\left(TN+FN\right)}}$$

## 5. Datasets

### 5.1. MRBrainS18 [61,62]

The image data for this challenge were collected at the UMC Utrecht using a 3T scanner (The Netherlands). T1-weighted, T1-weighted inversion recovery, and T2-FLAIR scans of 30 subjects have been fully annotated. Alzheimer’s patients, patients with dementia, Diabetes, and, as well as matched controls (with increased cardiovascular risk) with varying degrees of atrophy and white matter lesions (age > 50), were included in the study. The voxel sizes for all scans are 0.958 mm, 0.958 mm, and 3.0 mm. The N4ITK algorithm is used to correct the bias fields in the scans.

### 5.2. IBRS

The Internet Brain Segmentation Repository (IBSR) [63] encourages the advancement of segmentation methods and the evaluation of MRI brain images. There are eighteen subjects ranging in age from 7to 71). It is also worth noting that these data were subjected to the CMA’autoseg’bias field correction routines.

### 5.3. BRATS

A trained human expert manually annotated multi-contrast MRI scans of ten patients with low-grade glioma and twenty patients with high-grade glioma with two tumor labels [64,65]. Furthermore, the training data consist of simulated images of 25 high-grade and 25 low-grade glioma patients with the same 2 “ground truth” labels. The test images included 11 high-quality and 4 low-quality real-world cases and 10 high-quality and 5 low-quality simulated images.

### 5.4. ADNI

Alzheimer’s MRI images were taken from the ADNI (Alzheimer’s disease Neuroimaging Initiative) database [66,67]. The primary purpose of ADNI is to track the progress of the disease and study the variation in brain function and structure during the four stages of the disease. ADNI has a clinical record of patients between 55 and 90, including males and females. Patients have undergone all the tests at subsequent intervals. This project is for collecting the anatomic, diffusion, perfusion, and resting-state MRI images.

### 5.5. ATLAS

A 955 T1-weighted MRI scans are available in the Anatomical Tracing of Lesions after Stroke (ATLAS) dataset [68]. These scans are divided into training (n = 655 T1w MRIs with manually segmented lesion masks) and testing (n = 300 T1w MRIs only; lesion masks are not released). T1-weighted average structural template images from MNI152 standard space are used. The database contains lesion and scanner metadata in two.csv files. The LONI Probabilistic Brain Atlas (LPBA40) is a collection of anatomical maps of the brain that can be found in Atlas. These maps were created using data from 40 human volunteers’whole-head MRIs. Each MRI was manually delineated to identify 56 brain structures, most of which are located in the cortex.

### 5.6. CHASE_DB1

A child heart and health study in England (CHASE_DB1) [69] contains 28 color retina images with a resolution of 999 × 960 pixels taken from the left and right eyes of 14 school children for segmenting retinal vessels.

### 5.7. DRIVE

The fundus images in the Digital Retinal Images for Vessel Extraction (DRIVE) [70] dataset include 7 abnormal pathology instances. It contains 40 images in JPEG format. The dataset is equally split for training and testing. The images are taken from a Netherlands screening program for diabetic retinopathy.

### 5.8. STARE [71]

The dataset contains 20 eye fundus images with a resolution of 700 × 605. In addition, two sets of ground-truth vessel annotations are available. Six images in this dataset are normal, and 11 indicate ophthalmological disease.

### 5.9. RITE [72,73]

Based on the publicly accessible DRIVE database, the RITE (Retinal Images Vessel Tree Extraction) database was created to enable comparative investigations on the segmentation or categorization of arteries and veins using retinal fundus images. Like DRIVE, RITE has 40 images evenly divided into training and test subsets. A fundus image, a vascular reference standard, and an arteries/veins (A/V) reference standard are included for each set. Four different types of vessels are identified for the A/V reference standard based on the vessel reference standard using four different colors. The image of the fundus is in tif format. The A/V and vessel reference standards are also in the png file format.

### 5.10. CCAP IEEE Data Port [74]

It is obtained from the IEEE Data Port and consists of the following five distinct sets of lung CT images: Viral Pneumonia, COVID-19, Bacterial, Pneumonia, Normal lung, and Mycoplasma Pneumonia (MP).

### 5.11. SARS-CoV-2 CT-Scan Dataset [75]

It included 1252 CT scans from patients infected with the disease and 1230 CT scans from patients not infected, for a total of 2482 CT scans.

### 5.12. CHAOS [76]

CHAOS provides CT and MRI data from healthy subjects for single and multiple abdominal organ segmentation.

### 5.13. ISLES [77]

In ISLES 201,863 patients’ information was included for training, while 40 patients’ information was added for testing. Furthermore, the developed methods are tested on a 40-stroke research dataset.

### 5.14. TCGA [78]

The TCGA project produced a massive amount of genomic, epigenomic, transcriptomic, and proteomic data. Transcriptomics technologies are methods for studying an organism’s transcriptome, the sum of its RNA transcripts. A proteome is a collection of proteins made by an organism. This information has improved our ability to diagnose, treat, and prevent cancer.

### 5.15. MOD [79]

It is a data set of pathological images with 30 images from the following 7 organs: colon, stomach, prostate, liver, breast, kidney, and bladder. The images in the dataset have a resolution of 1000 × 1000, with a total of about 21,000 nuclei. Professional pathologists label the boundaries.

### 5.16. BNS [80]

BNS is a 512 × 512-byte breast cancer image data set with 33 HE-stained pathological images. There are also manually labelled nuclei (2754) with tissue data from seven TNBC patients.

### 5.17. Medical Segmentation Decathlon (MSD) [81]

This repository includes segmented images and masks of liver, pancreas, spleen, colon, lungs, brain, hippocampus, prostate, heart, and heptic vessels.

## 6. Implementation Details

NVIDIA Deep Learning GPUs offer high processing power for deep learning model training. A software development kit (SDK) called NVIDIA CUDA-X AI [82] is intended for researchers and developers creating deep learning models. It utilizes powerful GPUs and satisfies several industrial benchmarks, including MLPerf. Computer vision tasks, recommendation systems, and conversational AI are all developed for NVIDIA CUDA-X AI. The following functionalities are supported by libraries in the NVIDIA Deep Learning SDK:

- Deep learning primitives are pre-built building blocks that can be used to define training elements such as tensor transformations, activation functions, and convolutions;
- Deep learning inference engine, a runtime you may use to deploy models in real-world settings;
- GPU-accelerated transcoding and inference are made possible by deep learning for video analytics, which also offers a high-level C++ runtime and API;
- Linear algebra—uses GPU acceleration to provide functionality for BLAS (basic linear algebra subprograms). Compared to the CPU this is 6–17 times faster;
- Sparse matrix operations let to use of GPU-accelerated BLAS with sparse matrices, such asthose required for natural language processing (NLP);
- Multi-GPU communication—allows for group communications over up to eight GPUs, including broadcast, reduction, and all-gather.

Tensor flow [83,84] is a free and open-source end-to-end platform for performing machine learning tasks, and Keras [85,86] is a tensor flow-based neural network library at a high level.

## 7. Comparison of UNET with Other Encoder–Decoder Deep Learning Model

The encoder–decoder deep learning model to segment the medical images alternate to UNET are FCN, FPN, Segnet, and Deeplab. FCN is the first encoder-decoder model. The convolution layer in the FCN [87] is the 1 × 1 convolution, which classifies and creates the mask at the pixel level by upsampling the last convolution layer through the deconvolution layer. However, the global contextual information is not obtained in the FCN, which reduces its segmentation performance and does not tune the parameters according to the image’s content. FPN (feature pyramid network) transmits the feature’s gradient information from the encoder to the decoder through the skip connection [88]. The depth of the model and separate encoder in the FPN increase the computational complexity [89]. UNET outperforms the segnet by producing higher accuracy in the multi-class classification of the COVID-19 dataset [90]. In addition, the segmentation accuracy for segnet can be improved with UNET. For example, a patch-wise residual-based squeeze U-SegNet model can increase the segmentation accuracy of the brain MRI to segment the GM, WM, and CSF [91]. In Deeplab [92], spatial pyramid pooling is used to adapt the pooling operation according to the different input images. Dilated or atorous convolution and depth separable convolution are other building blocks in the deeplab model applied to consider the spacing between the pixels and reduce the convolutional operation for RGB input.

## 8. Discussion

There are many medical image processing performed using the deep learning technique. However, segmentation is of great interest in diagnosing diseases. UNET can be fine-tuned according to the application and still has significant advancement potential in application range, training speed optimization, feature enhancement and fusion, a small sample training set, and training accuracy. Modified architectures of U-Net have recently been used to achieve precise segmentation of different lesions by embedding attention mechanisms, dense modules, residual structures, and other modules. Choosing an efficient UNET model is challenging; hence, it is implemented for different datasets.Evaluation metrics and limitations of different models are discussed in Table 1. The computational time, learning rate, and contribution of each model are summarized in Table 2.

## 9. Conclusions and Future Work

Clinical applications and academic research are significantly influenced by the analysis and processing of medical data. Deep learning can generate novel concepts for medical image techniques that enable texture morphology detection purely from data. It has emerged as the primary component in numerous medical image research. The outcomes demonstrate that the DL approach on CNN has received widespread acclaim for its medical image segmentation, classification, and other areas. This article examines the evolution of UNET architecture for segmenting the region of interest from different internal organs. This review also specified the evaluation metrics and segmentation regions obtained from the UNET models according to the diseases. In future work, segmentation accuracy can be improved by increasing the segmentation validation metrics. UNET can be cascaded with GAN for synthesizing the medical images and can be utilized for efficiently segmenting, classifying, and synthesizing the images. The architecture of UNET can be modified to predict the statistical information from the segmented region.

## Figures and Tables

**Table 1:** Evaluation metrics and limitations of different UNET models.

Model	Type of Disease Diagnosed	Evaluation Metrics			Limitations
UNET with generalized pooling [17]	Tumor	For the BRATS 18 dataset DSC WT-0.839 TC-0.6594 ET-0.7341 PPV WT-0.9175 TC-0.6564 ET-0.8175 Sensitivity WT-0.7879 TC-0.7169 ET-0.7367	For the BRATS19 dataset DSC WT-0.8764 TC-0.7465 ET-0.7926 PPV WT-0.9079 TC-0.7667 ET-0.8801 Sensitivity WT-0.8697 TC-0.8568 ET-0.8167		Assigning the average initial weight to each element complicates the model.
Stack Multi-Connection Simple Reducing Net (SMCSRNet) [18]	Tumor	Dice score-0.831 PPV-0.73 Sensitivity-0.87			When stacking more basic blocks (after 10),the performance decreases, and the number of parameters continuously increases. Therefore, it does not perform well for enhanced tumors. However, it is the end-to-end model which predicts the entire image.
3D spatial weighted UNET [19]	Psychological changes in the brain with age.	DSC GM-86.58 ± 1.76% WM-89.87 ± 1.43% CSF-84.81 ± 2.33% HD GM-1.29 ± 0.25 WM-1.73 ± 0.50 CSF-1.84 ± 0.31 AVD(Absolutevolume difference) GM-5.75 ± 3.58 WM-5.47 ± 5.19 CSF-6.84 ± 4.14			It can be implemented only in the 3D input.
AnatomicallygatedUNET [20]	Alzheimer’s disease	ADNI DC-0.8864 ± 0.0212 ASD-0.386 ± 0.058	LONI DC-0.8067 ± 0.0383 ASD-1.070 ± 0.036		Two sub-networks increase the segmentation’s memory burden. The similarity between an atlas and a segmented MRI is not considered. Image intensity data is not included
MH-UNET [21]	Tumor, stroke	Tumor- DSC WT-90% TC-83% ET-78% HD WT-4.164 TC-9.809 ET-32.200	Stroke DSC-82% HD-17.69 Average Distance-0.68 Precision-77 Recall-0.37 AVD-5.61		During the segmentation of whole tumor, dice score will become zero.
MI-UNET [22]	Stroke	DC-56.72% HD-23.94 ASSD-7 Precision-65.45 Recall-59.38			The registration step occupies computational time. Difficult to segment the small lesions
Multi-Res Attention UNET [24]	Epilepsy	DC-76.62% Precision-87.97% Recall-67.09%			Attention gating signal should be optimally chosen to increase the recall rate
GLUE [26]	Ophthalmic diseases	ForDRIVE Dataset Accuracy-0.9692 Sensitivity-0.8278 Specificity-0.9861 Precision-0.8637	For STARE Dataset Accuracy-0.9740 Sensitivity-0.8342 Specificity-0.9916 Precision-0.8823		The model’s first part (WUN) has 23.49 M parameters, and the second part (WRUN) has 32.43 M parameters. Therefore, it has to be separately trained.
S-UNET [28]		For CHASE-DB1 dataset MCC-0.8065 SE-0.8044 SP-0.9841 Accuracy-0.9.58 AUC-0.9867 F1 score-0.8242	ForTONGREEN Dataset MCC-0.7806 SE-0.7822 SP-0.9830 Accuracy-0.9652 AUC-0.9824 F1 score-0.7994	For DRIVE dataset MCC-0.8055 SE-0.8312 SP-0.9751 Accuracy-0.9567 AUC-0.9821 F1 score-0.8303	Not applicable for Patch-based segmentation
UNET with atrous Separable [29]	Cancer	For MOD dataset Accuracy-92.82 ± 0.43 Precision-88.54 ± 0.58 Recall-86.46 ± 0.84 F1 score-87.35 ± 0.75 IoU-77.72 ± 1.15	For BNS dataset Accuracy-96.86 ± 0.26 Precision-88.29 ± 0.80 Recall-86.19 ± 0.67 F1 score-86.97 ± 0.1 IoU-77.31 ± 0.11		3.96 million parameters for sepconvolution with atrous and 1.01 million parameters without atrous.
RIC UNET [30]	Cancer	Aggregated Jaccard index-0.5635 Dice-0.8008 F1 score-0.8278			It has a more substantial discrimination effect on some deeper backgrounds.
Modified 2D UNET [31]	Coronary artery disease	Only aorta- DC-91.20% IoU-83.82%	Aorta with coronary artery DC-88.80% IoU-79.85%		Small regions of the proximal coronary artery are occasionally missed while using this model.Cannot produce high accuracy for segmenting aorta with coronary artery.
UCNET with attention Mechanism [32]	Cardiac arrhythmia and Congenital cardiac diseases	Single modality DSC-0.9112 Jaccard-0.8420	Multimodality DSC-0.91112		Attention mechanisms must be carefully selected for each task based on their characteristics
Cascaded UNET [33]	COVID-19	DSC-62.8%			The tradeoff between TPR and FPR rate.
Res-D-UNET [35]	Pulmonary embolism	For CT lung dataset DSC-0.982 Precision-0.985 Recall-0.980 SSIM-0.961	For CHAOS dataset DSC-0.969 Precision-0.966 Recall-0.968 SSIM-0.951		Hyper-parameters must be set through many experiments and adjustments.
Radiomics guided –DUN GAN [37]	Liver lesions	DSC-93.47 ± 0.83 Accuracy-96.23 Recall-91.79			Segmentor and discriminator have to be trained separately.
Dilated Dense attention UNET [40]	Esophageal tumor segmentation	DSC-0.79 ± 0.20, Mean surface distance-5.4 ± 20.2 mm 95% Hausdorff distance-14.7 ± 25.0 mm			Performance is worse for Smaller tumor cells (30cc), while patients with a disturbance in esophageal, hiatal hernia, proximal tumor had no discernible network strength.
HDRDS UNET [41]	Lymph node cancer	DSC-0.7811 SEN-0.9357 HMSD-0.8514			Only 60% of the training volumes are used in model selection, reducing the trained models’ generalization ability and validation performance.
HF-UNET [42]	Prostate cancer	DC-0.88 ASD-1.31 SEN-0.88 PPV-0.89			Choosing the information weight as 0 and 1 will degrade the late and dual branch network.

**Table 2:** Summary of Model.

References	Modification in UNET	Dataset		Area of Segmentation	Contributions	Computational Time
		Clinically Available Dataset	Publically Available Dataset			
[17]	Generalized pooling and adaptive weight.		BRAT 2018 and BRAT 2019	Brain	Extract valuable features during down-sampling. Generalized pooling is applied to varying data.	Learning rate is 0.0001.
[18]	Stacking three SRUNET. In total, 32 feature maps are added in the last UNET, stacked by a long skip connection to the input image.		BRAT2015		Reduces 4/5 parameters compared to the original UNET. Additionally, it reduces multi-scale feature fusion.	Learning rate-4 × 10^−5^. Epoch-12. This model takes 9.6 s to segment the tumor, and training time is 4 h 29 min (two stack level). Therefore, the learning rate is 4 × 10^−5^. Batch size is 10. Reduces the computational time.
[19]	A volumetric feature recalibration layer is included.		Multi-atlas Labeling (MIAL) MICCAI 2012 Grand Challenge		Spatial information loss can be avoided, and the power of the features can be enhanced.	This model s trained for 20,000 iteration with initial learning rate is 0.001. After that, the learning rate becomes half every 5000 iterations. It takes 1 day to train the model.
[20]	The anatomical gate learns the anatomical features from the brain atlases and guides the segmentation network for segmenting the correct region of interest.		ADNI and LONI-LPBA40		The feature map learned from the input image fuses with the multi-label atlases to increase segmentation performance.	It takes approximately one day to train the model. Learning rate-0.001, number of epoch is 1000, minibatch size-1.
[21]	Dense block, residual inception block, and hierarchical blocks are included		MICCAI BraTS and ISLES		Gradient vanishing and exploitation gets reduced. Less learnable parameter	For MICCAI BrasChallengedatase, The learning rate is 4 × 10^−5^. Batch size is 1. Epochs-300. For ISLES dataset, Initial learning rate 5 × 10^−4^, Epochs-300, batch size-4
[22]	The LDDMM algorithm performs brain parcellation.		ATLAS		It can be applied to all types of input regardless of the dimensions.	Learning rate 0.001. It takes 140 s to segment strokes. Batch size-32.
[24]	The chain of the 3 × 3 kernel is connected in series.	SCTIMST, Trivandrum, India.			Consider the large semantic gap feature map between encoder and decoder. It suppresses redundant features. It reduces higher memory requirements.	The learning rate is 0.0001
[26]	Weighted attention mechanism, and skip connection are added.		DRIVE and STARE dataset	Eye	Data imbalance reduced.	Learning rate is 5 × 10^−5^. (batch size 128). A number of epochs is 60. DRIVE dataset takes 91 minto train and STARE dataset takes 65 min to train the model. Segments the 20 retinal images within 6.2 s.
[28]	Two MI-UNET with saliency mechanism is included.	TONGREN	DRIVE, HASE_DB1		Data imbalance reduced.	DRIVE dataset-It takes 3 h to train the model and segment the vessel within 33 ms. TONGREN dataset-9 h for training and 0.49 s to segment. CHASE-DB1 dataset-5hours for trainingand 91 ms to segment
[29]	Convolutional operation is changed into sep convolution.		MOD and BNS	Cell or nuclei	Size, trainable parameter, and evolution time reduced.	The learning rate is 1 × 10^−3^. Epochs-50
[30]	Residual block, channel gate, and multi-scale are applied in UNET.		The Cancer Genomic Atlas		Extract the different cell shapes from the dense cell.	The learning rate is 0.0001, which is reduced by ten percent per 1000 iterations. Batch size is 2. Epoch-100
[31]	Batch normalization and dropout layer are added.	University College Hospital London and Barts Health NHS Trust.		Heart	Reduced overfitting and stabilized the training process.	The learning rate is 1 × 10^−5^. Epochs-200. Segmenting time is 40–141 s.
[32]	SNEM, attention mechanism, and clique UNET are included.	Cardiac CT angiography at Shuguang Hospital, Shanghai, China.			More salient features can focus.	Learning rate 0.001, drop out rate is 0.8. Epochs-80,000
[33]	Expectation maximization algorithm.	CT datasets from Iran, Italy, South Korea, and the United States from multiple institutions		Lung	Semantic label not required.	The learning rate is 0.0005
[35]	Residual and dense networks are embedded in UNET.	China-Japan Friendship Hospital,	CHAOS CT images		Attenuate the problem of degradation and vanishing gradient. Overfitting gets reduced.	Learning Rate 2 × 10^−4^ (batch size is 4). Running time 1096.7 s. Numberofepochs is 100.
[37]	Radiomics features, dense layer, and GAN are added.	McGill University Health Centre		Liver	Network converges faster and smoother.	The learning rate is 1 × 10^−6^ for segmentoranddiscriminator. Batch size is 2 for segmentor and 64 for discriminator.
[40]	Dilated dense spatial attention gate and channel attention gate are included.	Dataset approved by Leiden University Medical Center’s Medical Ethics Review Committee in The Netherlands		Esophageal	Receptive field increases without increasing the network size	Training time-6 days. Batch size 7
[41]	Hyper dense encoder and recurrent dense siamesedecoder are added.	General Hospital of Shenyang Military Area Command(F-FDG PET/CT Scan)		Lymphoma	Stable gradient, explore spatial-temporal correlation.	The initial learning rate is 0.001, and it will be halved after each 10,000 iterations. Validationof model is performedafter each 200 iterations.
[42]	The contour extracts the prostate region. Attention-based task consistency learning block learns the data from segmentation and regression.		National Cancer Institute—International Symposium on Biomedical Imaging (NCI-ISBI) 2013 Automated Segmentation of Prostate Structures Challenge dataset.	Prostate	Accurate contours are created to segment the prostate.	A number of epochs 60. The learning rate is decreased from 0.01 to 0.0001 by a step size of 2 × 10^−5^

## Data Availability

No new data were created or analyzed in this study. Data sharing is not applicable to this article.
